# Review on Zinc Oxide Nanoparticles: Antibacterial Activity and Toxicity Mechanism

**DOI:** 10.1007/s40820-015-0040-x

**Published:** 2015-04-19

**Authors:** Amna Sirelkhatim, Shahrom Mahmud, Azman Seeni, Noor Haida Mohamad Kaus, Ling Chuo Ann, Siti Khadijah Mohd Bakhori, Habsah Hasan, Dasmawati Mohamad

**Affiliations:** 1 Nano-Optoelectronics Research and Technology Laboratory (N.O.R. Lab), School of Physics, Universiti Sains Malaysia, 11800 Minden, Pulau Pinang Malaysia; 2 Advanced Medical and Dental Institute, Cluster of Integrative Medicine, Universiti Sains Malaysia, 13200 Bertam, Malaysia; 3 School of Chemical Sciences, Universiti Sains Malaysia, 11800 Minden, Pulau Pinang Malaysia; 4 Department of Medical Microbiology, Parasitology and Immunology, School of Medical Sciences, Universiti Sains Malaysia, Kubang Kerian, 16150 Kubang Kerian, Kelantan Malaysia; 5 School of Dental Sciences, Universiti Sains Malaysia, 16150 Kubang Kerian, Kelantan Malaysia

**Keywords:** Antibacterial activity, ZnO-NPs, Toxicity mechanism, Reactive oxygen species, Zinc ions release, Food antimicrobial

## Abstract

Antibacterial activity of zinc oxide nanoparticles (ZnO-NPs) has received significant interest worldwide particularly by the implementation of nanotechnology to synthesize particles in the nanometer region. Many microorganisms exist in the range from hundreds of nanometers to tens of micrometers. ZnO-NPs exhibit attractive antibacterial properties due to increased specific surface area as the reduced particle size leading to enhanced particle surface reactivity. ZnO is a bio-safe material that possesses photo-oxidizing and photocatalysis impacts on chemical and biological species. This review covered ZnO-NPs antibacterial activity including testing methods, impact of UV illumination, ZnO particle properties (size, concentration, morphology, and defects), particle surface modification, and minimum inhibitory concentration. Particular emphasize was given to bactericidal and bacteriostatic mechanisms with focus on generation of reactive oxygen species (ROS) including hydrogen peroxide (H_2_O_2_), OH^−^ (hydroxyl radicals), and O_2_^−2^ (peroxide). ROS has been a major factor for several mechanisms including cell wall damage due to ZnO-localized interaction, enhanced membrane permeability, internalization of NPs due to loss of proton motive force and uptake of toxic dissolved zinc ions. These have led to mitochondria weakness, intracellular outflow, and release in gene expression of oxidative stress which caused eventual cell growth inhibition and cell death. In some cases, enhanced antibacterial activity can be attributed to surface defects on ZnO abrasive surface texture. One functional application of the ZnO antibacterial bioactivity was discussed in food packaging industry where ZnO-NPs are used as an antibacterial agent toward foodborne diseases. Proper incorporation of ZnO-NPs into packaging materials can cause interaction with foodborne pathogens, thereby releasing NPs onto food surface where they come in contact with bad bacteria and cause the bacterial death and/or inhibition.

## Introduction

Nanotechnology is a research hot spot in modern materials science. This technology is capable of providing miscellaneous novel applications that range from innovative fabric compounds, food processing, and agricultural production to sophisticated medicinal techniques [1]. It is considered as the synthesis, characterization, and exploration of materials in the nanometer region (1–100 nm). At this level, the properties and functions of living and anthropogenic systems are defined [2]. In this technology, the pertinent materials are those whose structures exhibit new and considerably enhanced physicochemical and biological properties as well as distinct phenomena and functionalities as a result of the nanoscale size [3]. This nanoscale size generally confers larger surface areas to nanoparticles (NPs) compared with macro-sized particles [4]. NPs are known as controlled or manipulated particles at the atomic level (1–100 nm). They show size-related properties significantly different from bulk materials [5]. Given their small size, NPs have larger structures in comparison with their counterparts. This distinct property allows their possible applications in many fields such as biosensors, nanomedicine, and bionanotechnology [4]. The intrinsic properties of metal NPs such as zinc oxide (ZnO), TiO_2_, and silver are mostly characterized by their size, composition, crystallinity, and morphology. Reducing the size to nanoscale can modify their chemical, mechanical, electrical, structural, morphological, and optical properties. These modified features allow the NPs to interact in a unique manner with cell biomolecules and thus facilitate the physical transfer of NPs into the inner cellular structures [6]. Nanostructured materials have a larger percentage of atoms at their surface which lead to high surface reactivity. Thus, nanomaterials have witnessed recently significant importance in the basic and applied sciences as well as in bionanotechnology.

Nano-sized ZnO exhibits varying morphologies and shows significant antibacterial activity over a wide spectrum of bacterial species explored by a large body of researchers [5, 7–13]. ZnO is currently being investigated as an antibacterial agent in both microscale and nanoscale formulations. ZnO exhibits significant antimicrobial activities when particle size is reduced to the nanometer range, then nano-sized ZnO can interact with bacterial surface and/or with the bacterial core where it enters inside the cell, and subsequently exhibits distinct bactericidal mechanisms [10]. The interactions between these unique materials and bacteria are mostly toxic, which have been exploited for antimicrobial applications such as in food industry.

Interestingly, ZnO-NPs are reported by several studies as non-toxic to human cells [14], this aspect necessitated their usage as antibacterial agents, noxious to microorganisms, and hold good biocompatibility to human cells [12]. The various antibacterial mechanisms of nanomaterials are mostly attributed to their high specific surface area-to-volume ratios [15], and their distinctive physicochemical properties. However, the precise mechanisms are yet under debate, although several proposed ones are suggested and adopted. Investigations on antibacterial nanomaterials, mostly ZnO-NPs, would enhance the research area of nanomaterials, and the mechanisms and phenomenon behind nanostructured materials.

Bacterial infectious diseases are serious health problem that has drawn the public attention in worldwide as a human health threat, which extends to economic and social complications. Increased outbreaks and infections of pathogenic strains, bacterial antibiotic resistance, emergence of new bacterial mutations, lack of suitable vaccine in underdeveloped countries, and hospital-associated infections, are global health hazard to human, particularly in children. For example, infections by *Shigella flexneri* cause 1.5 million deaths annually, due to contaminated food and drinks by these bacteria [16]. Thus, developing novel antibacterial agents against bacteria strains, mostly major food pathogens, such as *Escherichia coli* O157:H, *Campylobacter jejuni*, *Staphylococcus aureus*, *Pseudomonas aeruginosa*, *Enterococcus faecalis*, *Salmonella* types, and *Clostridium perfringens*, has become utmost demand. This work is intended to explore these problems to induce further investigations in these areas by addressing new techniques, benefiting from the unique features of ZnO-NPs, and from to date successful studies.

In this paper, we have extensively reviewed ideas behind the antibacterial activity of ZnO-NPs covering techniques of evaluating bacteria viability. In the subsequent sections, we have discussed the factors affecting the antibacterial activity, including UV illumination, ZnO particle size, concentration, morphology, surface modifications by annealing, surface defects, and the minimum inhibitory concentration (MIC) and minimum bactericidal concentration (MBC). A brief presentation of an experimental case study, carried by authors on antibacterial activity response to *E. coli*, was explored. A special focus has been given on a range of remarkable toxicity mechanisms that underlie this bacterial activity, mainly reactive oxygen species (ROS) generation and Zn^2+^ release. Finally, a concise discussion was made to an essential application of ZnO-NPs antibacterial activity as an antimicrobial agent against foodborne diseases and food packaging.

## Zinc Oxide Nanoparticles

ZnO is described as a functional, strategic, promising, and versatile inorganic material with a broad range of applications. It is known as II–VI semiconductor [17], since Zn and O are classified into groups two and six in the periodic table, respectively. ZnO holds a unique optical, chemical sensing, semiconducting, electric conductivity, and piezoelectric properties [18]. It is characterized by a direct wide band gap (3.3 eV) in the near-UV spectrum, a high excitonic binding energy (60 meV) at room temperature [19–23], and a natural n-type electrical conductivity [24]. These characteristics enable ZnO to have remarkable applications in diverse fields [20]. The wide band gap of ZnO has significant effect on its properties, such as the electrical conductivity and optical absorption. The excitonic emission can persevere higher at room temperature [21] and the conductivity increases when ZnO doped with other metals [19]. Though ZnO shows light covalent character, it has very strong ionic bonding in the Zn–O. Its longer durability, higher selectivity, and heat resistance are preceded than organic and inorganic materials [12]. The synthesis of nano-sized ZnO has led to the investigation of its use as new antibacterial agent. In addition to its unique antibacterial and antifungal properties, ZnO-NPs possess high catalytic and high photochemical activities. ZnO possesses high optical absorption in the UVA (315–400 nm) and UVB (280–315 nm) regions which is beneficial in antibacterial response and used as a UV protector in cosmetics [25].

### Synthesis of ZnO Nanostructures

ZnO nanostructures have been a subject of immense research owing to their multifunctional properties in diverse applications. The nanostructured ZnO has been emerged as a potential candidate for applications in sensors, energy harvesting, and many electronic devices. Many pronounced applications are being currently explored in the biomedical and antiviral areas. This is as a result of their potential biocompatibility over other metal oxides, solubility in alkaline medium, and the Zn–O terminated polar surfaces [26]. The unique properties and versatility of ZnO pave the way to use various methods to synthesize various ZnO nanostructures. ZnO-NPs can be synthesized through various methods by controlling the synthesis parameters. The properties can be tailored by shape and size, resulting in renewable applications relevant to their structural properties. Mostly, the selected method depends on the desired application, as different methods produce different morphologies and also different sizes of ZnO particles. Accordingly, the chemical and physical parameters such as the solvent type, precursors, pH, and the temperature were highly considered. An assortment of ZnO nanostructures with different growth morphologies such as nanorods, nanosphere, nanotubes, nanowires, nanoneedles and nanorings have been successfully synthesized [27]. Such unique ZnO nanostructures reflected the richest nanoconfiguration assembly compared to other nano-metal oxides, in terms of properties and structure, such as nanobelts, nanocages, nanocombs and nanosprings/nanohelixes [19]. Other shapes can also be obtained, such as ZnO spirals, drums, polyhedrons, disks, flowers, stars, boxes, and plates [28], those are possibly grown by adjusting the growth conditions. Each nanostructure has specific structural, optical, electrical, and physicochemical properties [29], permitting remarkable applications. These nanostructures have been fabricated using variety of physical and chemical techniques; however, the chemical techniques allow better control of the particle size and morphology [30]. The most adopted fabrication methods include thermal evaporation of ZnO powders at 1400 °C, hydrothermal synthesis, sol–gel technique, simple thermal sublimation, self-combustion, polymerized complex method, vapor–liquid–solid technique, double-jet precipitation, and solution synthesis [27, 31]. The solution process was used by several researchers to produce selective ZnO nanostructures. Wahab et al. [32] have synthesized flower-shaped ZnO nanostructures which were produced via solution process at low temperature (90 °C) using the zinc acetate dihydrate and NaOH. As well, Zhang et al. [33] have synthesized the flower-shaped, prism, snowflakes, and rod-like morphologies ZnO, at a high temperature of 180 °C for 13 h. The researchers also prepared prism-like and prickly sphere-like ZnO via decomposition process at 100 °C for 13 h. These nanostructures plus others such as nanowires, nanoplates, and nanorods have been key factors for the antibacterial activity, as each morphology accounts for a certain mechanism of action. Thus, a large number of researchers have been motivated to achieve selective nanostructured ZnO for the antibacterial tests. They succeeded to produce morphologies that were highly compatible with the antibacterial activity. Wahab et al. [34] carried a non-hydrolytic solution process using zinc acetate dihydrate to prepare ZnO-NPs. The method yielded structures of spherical surface that showed high antibacterial activity against the tested pathogens. Similarly, spherical shaped ZnO-NPs in another investigation [35] were obtained via soft chemical solution process, and it was used for the treatment of bacteria (*E. coli*, *S. aureus*, *P. aeruginosa*, *B. subtilis*, and *S. acidaminiphila*) and cancer cells (HepG2 and MCF-7 cell lines). While Stanković et al. [36] have synthesized ZnO powder hydrothermally with the addition of different stabilizing agents leading to different nanostructures. The obtained synthesized ZnO has shown nanorods of hexagonal prismatic and hexagonal pyramid-like structures, with some spherical and ellipsoid shapes. These different morphologies displayed pronounced antibacterial effect toward the targeted bacteria. Further discussions are in Sect. 4.2 coupled with Table 1 displaying some synthesis methods and their corresponding morphology of ZnO.

**Table 1:** ZnO synthesis and resultant morphology

Techniques	ZnO morphology	References
Microwave decomposition	Sphere	Jalal et al. [9]
Simple wet chemical route	Nano and micro-flowers, dumbbell shaped, rice flakes, and rings	Ramani et al. [129]
Deposition process	Dumbbell- and rod-shaped ZnO	
Simple precipitation method	Nano-flakes	Kumar et al. [146]
Hydrothermal synthesis	Hexagonal prismatic rods	Stanković et al. [36]
Solvothermal method	Nano-flowers, nanorods, nano-spheres	Talebian et al. [77]
Microwave hydrothermal method	Mulberry-like	Ma et al. [78]
Hydrothermal technique	Nanorods	Hafez et al. [147]

In a more recent study [37], ZnO nanowires were synthesized in heterojunction of silver-loaded ZnO (Ag_*x*_ Zn_1−*x*_O–ZnO nanowires) through UV light decomposition process. It was found to exhibit higher antibacterial activities to *E. coli* under visible light or in the dark. It disrupted the bacterial membrane and released lethal active species.

Techniques of doping and implanting foreign metals on ZnO nanostructures to develop functional antibacterial agent have become a topic among researchers. Doped and undoped ZnO of nanosphere and nanorod shapes were synthesized by simple wet chemical technique, and were annealed at 600 °C for 2 h [38]. The resultant ZnO samples were tested against three bacterial strains (*E. coli*, *P. aeruginosa*, and *S. aureus*). ZnO-doped samples exhibited considerably high activity toward *S. aureus* (skin bacteria) in comparison to *E. coli* and *P. aeruginosa*. It produced zone of inhibition of 4 % which was 37 % higher than that produced by undoped ZnO nanostructures. These results were beneficial for medical application. *S. aureus* is well known of causing contamination in hospital implants leading to serious infections [39]. ZnO is characterized by its antibacterial coatings, incorporation in skin creams, and UV protection. Therefore, coating hospital implants with 4 % of this doped ZnO nanostructures will be more effective in controlling associated bacterial infections. On the other hand, such doped ZnO can alternatively be used in skin lotions and in UV protection than undoped ZnO.

Usually, antibacterial tests are done in aqueous media or cell culture media. ZnO is known as nearly insoluble in water, it agglomerates immediately with water during synthesis due to the high polarity of water leading to deposition. Issues of aggregation, re-precipitation, settling, or non-dissolution impede the synthesis processes. In this regards, a number of researchers considered this difficulty by using certain additives that have no significant effect in the antibacterial activity. As such, in the above mentioned study [36], the addition of PVA, polyvinylpyrrolidone (PVP), and poly(α, γ, l-glutamic acid) (PGA; see Abbreviations) as stabilizers enhanced ZnO morphology and size for the antibacterial activity. Meanwhile, Zhang et al. have addressed the problem by adding dispersants polyethylene glycol (PEG; see Abbreviations) and PVP (10 % of the amount of ZnO-NPs) which enhanced the stability of ZnO and resulted in ZnO nanofluids, well suited for the antibacterial tests. While other researchers used appropriate capping agents [12] or deflocculants (sodium silicate Na_2_SiO_3_ or sodium carbonate Na_2_CO_3_). After addition, the mixtures were exposed to vigorous vortex (e.g., 5 min) or kept overnight on magnetic stirring and then ultrasonicated 20–30 min to avoid aggregation and deposition of particles. Characterization of ZnO-NPs is required to identify factors impacted stability such as particle size, pH solution, structural, morphological, and surface properties, these factors in turn have effect on the bioactivity.

### Crystal Structure of ZnO

**Fig. 1:**
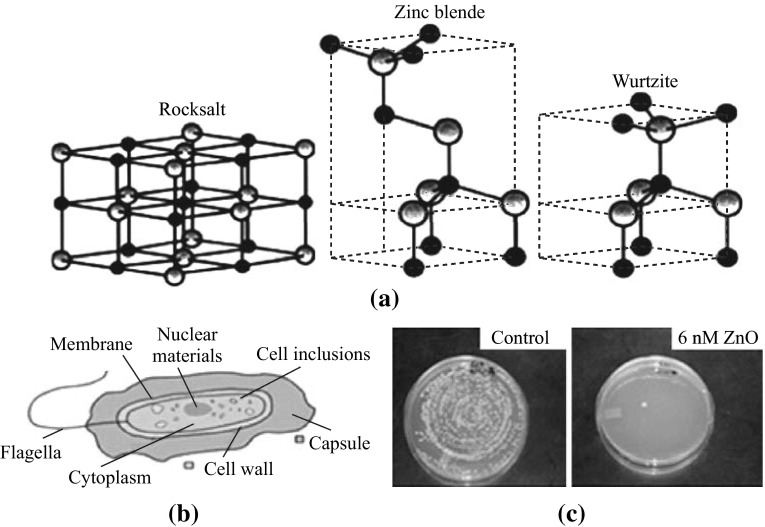
**a** ZnO crystal structures. Adapted from Ozgur et al. [41]. **b** Bacterial cell structures, reused from Earth Doctor, Inc., formerly Alken-Murray [45]. **c** *S. aureus* plating for colony count [13]

ZnO exhibits three crystallize structures namely, wurtzite, zinc-blende and an occasionally noticed rock-salt [41, 42]. The hexagonal wurtzite structure possesses lattice spacing *a* = 0.325 nm and *c* = 0.521 nm, the ratio *c*/*a* ~ 1.6 that is very close to the ideal value for hexagonal cell *c*/*a* = 1.633. Each tetrahedral Zn atom is surrounded by four oxygen atoms and vice versa [43]. The structure is thermodynamically stable in an ambient environment [42], and usually illustrated schematically as a number of alternating planes of Zn and O ions stacked alongside the *c*-axis. Zinc-blende structure is metastable and can be stabilized via growth techniques. These crystal structures are illustrated in (Fig. 1a), and the black and gray-shaded spheres symbolize O and Zn atoms, respectively.

## Antibacterial Activity of ZnO Nanoparticles

Bacteria are generally characterized by a cell membrane, cell wall, and cytoplasm. The cell wall lies outside the cell membrane and is composed mostly of a homogeneous peptidoglycan layer (which consists of amino acids and sugars). The cell wall maintains the osmotic pressure of the cytoplasm as well the characteristic cell shape. Gram-positive bacteria have one cytoplasmic membrane with multilayer of peptidoglycan polymer [44], and a thicker cell wall (20–80 nm). Whereas gram-negative bacteria wall is composed of two cell membranes, an outer membrane and a plasma membrane with a thin layer of peptidoglycan [44] with a thickness of 7–8 nm. NPs size within such ranges can readily pass through the peptidoglycan and hence are highly susceptible to damage. The cytoplasm, a jelly-like fluid that fills a cell, involves all the cellular components except the nucleus. The functions of this organelle include growth, metabolism, and replication. Consequently, the cytoplasm contains proteins, carbohydrates, nucleic acids, salts, ions, and water (∼80 %). This composition contributes in the electrical conductivity of the cellular structure. The overall charge of bacterial cell walls is negative. Figure 1b shows typical bacteria cell structures [45]. Antibacterial activity is known according to *The American Heritage Medical Dictionary 2007*, as the action by which bacterial growth is destroyed or inhibited. It is also described as a function of the surface area in contact with the microorganisms [46]. While antibacterial agents are selective concentration drugs capable to damage or inhibit bacterial growth and they are not harmful to the host. These compounds act as chemo-therapeutic agents for the treatment or prevention of bacterial infections (*Saunders Comprehensive Veterinary Dictionary 2007*). An antibacterial agent is considered as bactericidal if it kills bacteria or as bacteriostatic if it inhibits their growth.

Different methods have been adopted for the assessment and investigation of antibacterial activity in vitro. These methods include disk diffusion, broth dilution, agar dilution, and the microtiter plate-based method [47]. Other methods are different according to the investigated parameters. For example, the conductometric assay measures the bacterial metabolism-induced alterations in the electrical conductivity of growth media [48, 49]. Meanwhile, Reddy et al. [50] have used the flow cytometry viability assays to examine ZnO-NPs toxicity toward *E. coli* and *S. aureus*. The most commonly used method is the broth dilution method, followed by colony count, through plating serial culture broths dilutions which contained ZnO-NPs and the targeted bacteria in appropriate agar medium and incubated. A number of researchers [13] have examined the antibacterial activity of ZnO-NPs to determine bacterial growth through the culture turbidity and the viable cells percentage by the colony counts test (Fig. 1c). While others, such as Yamamoto [51] enhanced the antibacterial activity of ZnO-NPs by modulating within the procedure. They considered that the antibacterial activity rate was much improved by decreasing the initial number of bacterial cells from 10^2^ to 10^6^ colony forming unit (CFU). Nair et al. [52] considered that the determination of starting number of bacterial cells is very important in the antibacterial activity evaluation. The MIC of an antimicrobial agent and MBC can be measured by using the susceptibility test methods. However, there are some variations in the established laboratory methods and protocols in the assessment of the bactericidal activity [53]. The agar diffusion method (an indirect method) is the most frequently used method and has been standardized as an official method for detecting bacteriostatic activity by the (ATCC). Other direct test methods, such as the measurement of urease inhibition of inocula, have been reported [54]. The microdilution method is a modification of the broth macrodilution test, which utilizes the advances in miniaturization to allow multiple tests to be performed on a 96-well plate. Modified procedures along with the standard methods are also used by a large body of researchers. In all of the aforementioned methods, the culture media [trypticase soy broth (TSB), Luria–Bertani broth (LB), nutrient agar (NA), tryptic soy agar (TSA), and blood agar (BA; see Abbreviations)] were accordingly selected to autoclave and stored at 4–5 °C. The stocks of ZnO-NPs suspensions are also usually prepared, and serially diluted to different concentrations, and then characterized using techniques [X-ray diffraction (XRD), field emission scanning electron microscope (FESEM), transmission electron microscope (TEM), energy dispersive X-ray spectroscopy (EDX), electron spectroscopy imaging (ESI), etc.; see Abbreviations] to correlate the antibacterial response with ZnO properties.

Growth curves were typically obtained via monitoring the optical density (OD), at wavelength of 600 nm, a typical wavelength for cells. The density of bacterial isolates must be adjusted to an optimal density of 0.5 McFarland standards. The OD should serially be monitored hourly up to 12 h of incubation, and finally after 24 h of overnight incubation for the determination of the percentage of growth inhibition [8]. The inhibition rate varies with the tested organisms and the utilized NP-oxide [55].

**Fig. 2:**
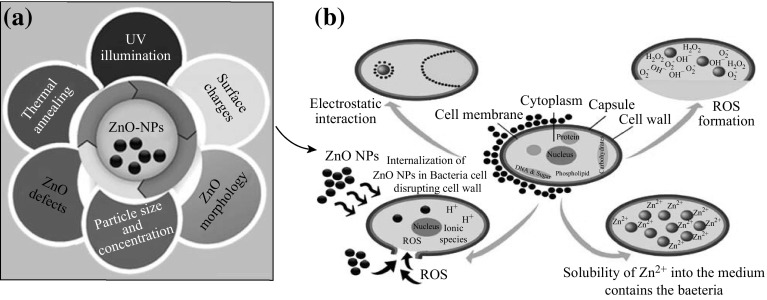
Correlation between the **a** influence of essential ZnO-NPs parameters on the antibacterial response and the **b** different possible mechanisms of ZnO-NPs antibacterial activity, including: ROS formation, Zn^2+^ release, internalization of ZnO-NPs into bacteria, and electrostatic interactions

We discuss below the influence of essential physiochemical and structural factors (Fig. 2a), which affect the antibacterial activity of ZnO-NPs, and consequently have potential impact upon the resultant toxicity mechanism (Fig. 2b).

## Mechanism of Antibacterial Activity of ZnO-NPs

Researchers analyzing morphological bacterial changes are induced by ZnO using SEM or FESEM to quantify the multiple mechanisms. Though, the antibacterial activity of ZnO-NPs has been referred to a number of issues, but the exact toxicity mechanism is not completely illuminated and still controversial, as there are some queries within the spectrum of antibacterial activity requiring deep explanations. Distinctive mechanisms that have been put forward in the literature are listed as following: direct contact of ZnO-NPs with cell walls, resulting in destructing bacterial cell integrity [7, 40, 56], liberation of antimicrobial ions mainly Zn^2+^ ions [57–59], and ROS formation [9, 60–62]. However, the toxicity mechanism varies in various media as the species of dissolved Zn may change according to the medium components besides the physicochemical properties of ZnO-NPs [59].

### UV Illumination Effect

ZnO is found to possess high photocatalytic efficiency among all inorganic photocatalytic materials, and is more biocompatible than TiO_2_ [63]. ZnO can highly absorb UV light [64], and it has a better response to UV light, thus its conductivity dramatically enhances, and this feature significantly activates the interaction of ZnO with bacteria. Its photoconductivity persists long after turning off the UV light, and it has been attributed to surface electron depletion region strongly associated to negative oxygen species (${\text{O}}_{2}^{ - } ,\;{\text{O}}_{2}^{2 - }$), adsorbed on the surface [65]. UV illumination rapidly causes desorption of this loosely bound oxygen from the surface. This results in reducing the surface electron depletion region and causing improved photoconductivity [66]. The photocatalysis is described as a photo-induced oxidation process that can damage and inactivate organisms [67]. ZnO-NPs in aqueous solution under UV radiation have phototoxic effect that can produce ROS such as hydrogen peroxide (H_2_O_2_) and superoxide ions (O^2−^). Such species are extremely essential for bio-applications [68]. The generated active species are capable to penetrate into the cells, thus inhibit or kill microorganisms. This process inspired the use of the photocatalytic activity of ZnO-NPs in bionanotechnology and in bionanomedicine for many antibacterial applications. Therefore, enhancement of ZnO bioactivity was considered as a result of the produced free radicals, as ZnO absorbs UV light [15]. A detailed reaction mechanism which explains this phenomenon was proposed by Seven et al. [69] and Padmavathy and Vijayaraghavan [12] as follows.

The electronic band structure of ZnO, as a semiconductor material consists of a conduction band (CB) and a valence band (VB). Incident radiation with photons of energy greater than 3.3 eV is immediately absorbed, thus the electrons move from the VB to the CB. This electron transfer initiates a series of possible photoreactions. As a result, positive holes (h^+^) are formed in the VB, while free electrons are created within the CB [69–71]. This positive hole (h^+^), a direct oxidant and essential for the creation of reactive hydroxyl radicals (OH^•^), serves as principal oxidants in the photocatalytic system [22, 70]. The electrons in the CB reduce oxygen, which is adsorbed by the photocatalyst [22]. Meanwhile, Padmavathy et al. proposed an association between photon reaction and the antibacterial activity in a series of reactions resulting in production of hydrogen peroxide (H_2_O_2_) molecules which penetrates the membrane, causing fatal damage. Sawai et al. [60] also attributed the disruption of the cell membrane to peroxidation of the unsaturated phospholipids as a result of photocatalytic prompted H_2_O_2_.

In antibacterial tests that involve UV exposure, OD readings are taken before and after UV illumination. Shorter exposures (15 min) result in significantly few colonies, while many colonies could be detected and counted by long exposure times (up to 30 min). Generally, it has been observed from the intensive studies held, that the antibacterial activity of ZnO can be verified under UV light as well as in the dark to inhibit the bacterial growth. Zhou et al. [72] reported best results of antibacterial effects upon UV exposure toward *E. coli* and *S. aureus*, which were 98.65 and 99.45 % under UV, respectively. The authors referred that to OH production under light, and they produced a novel ZnO complex. Their findings also showed that the activity can be achieved under UV illumination, ambient light, or even in the dark. However, ZnO exhibits considerable activity against bacteria under different test conditions [8, 56, 73] and fungi [61]. Besides, the active species that drive the activity can be created without UV irradiation [61, 74]. We have also carried a study [75] for the antibacterial activity toward *E. coli* and *S. aureus* using ZnO of two forms (ZnO-rod and ZnO-plate) which are exposed to UVA illumination (390 nm, 1.8 W cm^−2^). We found that UVA illumination had significantly influenced the interaction of both ZnO samples with the targeted bacteria compared with unexposed ZnO. Exposure of only 20 min increased the inhibition of *E. coli* by 18 % (ZnO-rod) and 13 % (ZnO-plate), whereas, for *S. aureus*, 22 % increase for treated with ZnO-rod and 21 % with ZnO-plate, compared with the unexposed. Thus, longer durations of UVA exposure expected to lead to greater growth inhibition. To elucidate the effects of oxygen species on antibacterial response, electrical, structural, and optical characterization of ZnO were performed. For example, the result of the current–voltage measurements (IV) showed significant increase in surface conductance (7-fold for ZnO-rod and 5-fold for ZnO-plate) due to a decrease in the depletion layer upon UVA illumination. It was suggested that photo-excitation caused desorption of the oxygen molecules from the surface, thus the surface potential decreased and underlying photoconductivity of ZnO. Stimulation of oxygen species such as H_2_O_2_, ${\text{O}}_{2}^{ - }$ and OH^•^ by UV light found to harm bacteria and damage the active enzyme, DNA, and protein [74, 76]. Our results were in consistence with Raghupathi et al. [13], whose results also revealed high antibacterial activity upon UV illumination.

### Impact of ZnO Morphology

The impact of ZnO shapes has attracted current research [3]. Many studies have reported that the toxicity is significantly affected by the various morphologies of ZnO-NPs [36, 77, 78]. ZnO morphology is determined by the synthesis conditions as mentioned earlier. Thus, desired synthesized ZnO-NPs structures for best antibacterial response could be attained by controlling parameters such as solvents, precursor types, and physicochemical settings such as temperature and pH [71] as well as shape-directing agents. Also, under controlled growth conditions, the surface morphology is determined by the surface activity.

The shape-dependent activity was explained in terms of the percent of active facets in the NPs. Synthesis and growth techniques lead to holding numerous active facets in NP. Rod-structures of ZnO have (111) and (100) facets, whereas spherical nanostructures mainly have (100) facets. High-atom-density facets with (111) facets exhibit higher antibacterial activity [3]. The facet-dependent ZnO antibacterial activity has been evaluated by few studies, and nanostructured ZnO with different morphologies have different active facets, which may lead to enhanced antibacterial activity [79]. In this regard, the shape of ZnO nanostructures can influence their mechanism of internalization such as rods and wires penetrating into cell walls of bacteria more easily than spherical ZnO-NPs [80]. Whereas, flower-shaped have revealed higher biocidal activity against *S. aureus*, and *E. coli* than the spherical and rod-shaped ZnO-NPs [77]. In addition to the enhancement of internalization, it has been suggested regarding the contribution of the polar facets of ZnO nanostructured to the antibacterial activity, that the higher number of polar surfaces possess higher amount of oxygen vacancies. Oxygen vacancies are known to increase the generation of ROS and consequently affect the photocatalytic of ZnO [81]. Currently, it has been found that greater antibacterial results could be achieved from ZnO morphologies of highly exposed (0 0 0 1)-Zn terminated polar facets [82].

### Surface Modification by Thermal Annealing

Functionalized ZnO surface leads to best antibacterial responses. Annealing of ZnO powder has much effect in increasing the inhibition. In our study [75], the EDX and IV measurements revealed that oxygen annealing increased the amount of oxygen atoms on the surface of ZnO samples. Oxygen annealing stimulated a high amount of oxygen atoms to be absorbed onto ZnO surface, thereby enhanced antibacterial response inducing more ROS in the suspension resulting in intense oxidative stress towards the bacteria. This was also in agreement with our study [83] that used ESI method to explore the zinc and oxygen atoms on ZnO structure, and it has shown a considerable increase of O:Zn ratio of the oxygen annealed samples. Modifying ZnO-NPs surface area would establish the release of Zn^2+^ ions and enhance ROS production. Mamat et al. [84] increased the surface area of ZnO nanorods by annealing under oxygen and air to stimulate formation of nanoholes on the surface to increase the surface area, and on turn have caused high absorption and diffusion of oxygen molecules onto the surface upon UV light exposure, which consequently assisted in generating more ROS on the surface.

1 $${\text{O}}_{2} \;({\text{g}}) + {\text{e}}^{ - } = {\text{O}}_{2}^{ - } \;({\text{ad}}),$$

2 $${\text{h}}\upnu \to {\text{e}}^{ - } + {\text{h}}^{ + } ,$$

3 $${\text{O}}_{2}^{ - } \;({\text{ad}}) + {\text{h}}^{ + } = {\text{O}}_{2} \;({\text{g}}).$$

An alternative way for surface modification can be attained by coating NPs with surface modifying reagents which trigger toxicity to bacteria, and can cause differences in Zn^2+^ ions release and ROS generation [85]. Hsu et al. [86] as well, completed an investigation on how the different surfactant molecules can result in varying antibacterial properties of ZnO-NPs.

### Influence of ZnO Particle Size and Concentration

**Fig. 3:**
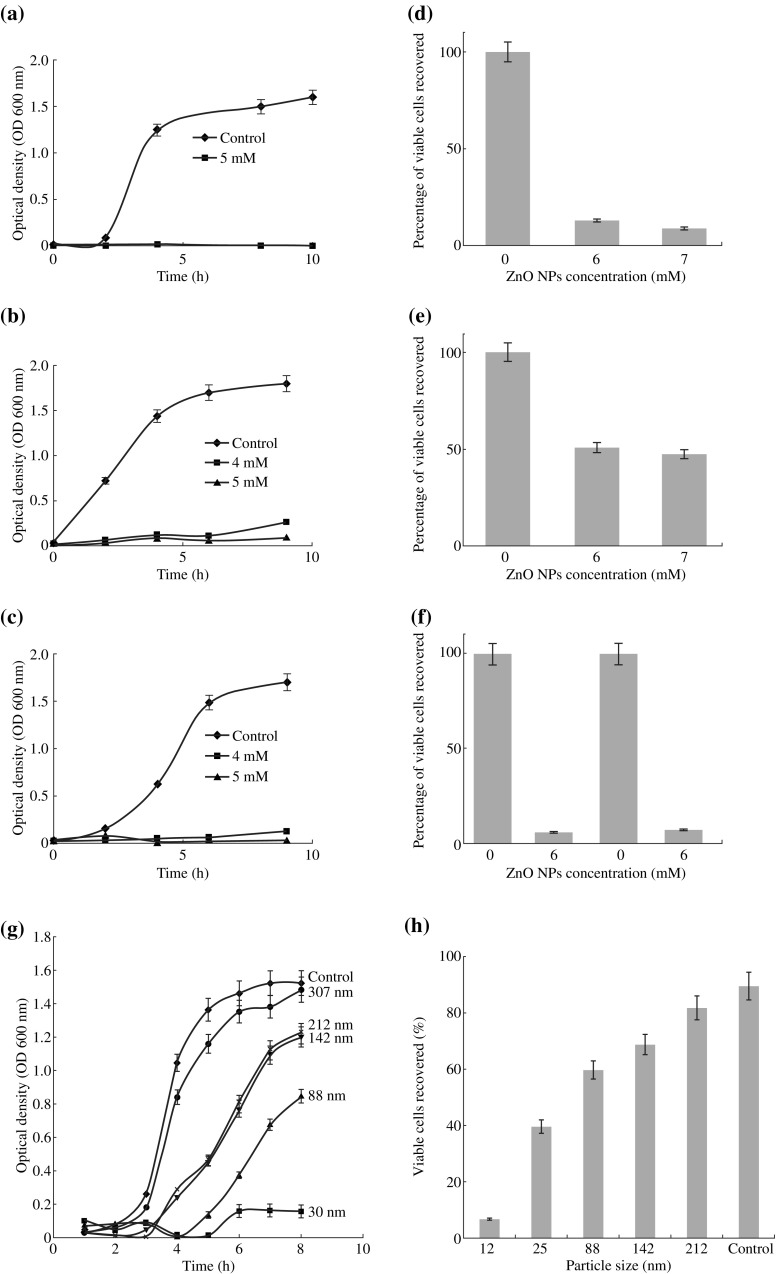
**a**–**f** Growth analysis curves and cells viability percentage, at selected ZnO concentrations. **g** Growth curves through optical density (OD_600 nm_) measurements. **h** Percentage of viable cells after overnight incubation. Adapted with permissions from Raghupathi et al. [13]

Particle size and concentration of ZnO-NPs play important roles in the antibacterial activity. ZnO-NPs antibacterial activity directly correlates with their concentration as reported by several studies, likewise, the activity is size dependent, however, this dependency is also influenced by concentration of NPs. Larger surface area and higher concentration are accountable for ZnO-NPs antibacterial activity [40, 87]. ZnO-NPs of smaller sizes can easily penetrate into bacterial membranes due to their large interfacial area, thus enhancing their antibacterial efficiency. A large number of studies investigated on the considerable impact of particle size on the antibacterial activity, and the researchers found that controlling ZnO-NPs size was crucial to achieve best bactericidal response, and ZnO-NPs with smaller size (higher specific surface areas) showed highest antibacterial activity [40, 51, 88]. The dissolution of ZnO-NPs into Zn^2+^ was reported as size dependent, and few studies suggested this dissolution of Zn^2+^ responsible for toxicity of ZnO-NPs. The effect of size and concentration was successfully analyzed by a work carried by Padmavathy and Vijayaraghavan [12] who described the generation of H_2_O_2_, which depends mainly on the surface area of ZnO. The larger the surface area and the higher concentration of oxygen species on the surface can obtain greater antibacterial activity by smaller particles, which was in contrast to that of Franklin et al. [89] who found no size-related effect. In general, a correspondence between NPs size and bacteria appears to be required for the bioactivity of ZnO-NPs, as well the concentration. Yamamoto [51] examined the influence of ZnO-NPs size (100–800 nm) on the antibacterial activity, against *S. aureus* and *E. coli* by changing the electrical conductivity with bacterial growth. It was concluded that decrease in particle size will increase the antibacterial activity. Similarly, it was found that ZnO-NPs antibacterial activity toward *S. aureus* and *E*. *coli* increases with decreasing the size [8, 12, 40]. Size-dependent bactericidal activity was also extensively evaluated by Raghupathi et al. [13]. The authors targeted a number of major gram-negative and gram-positive strains, and revealed that ZnO-NPs antibacterial activity was inversely proportional to the particle size. Based on the growth curves and percentage viability, their findings revealed that the activity is size dependent, where smaller sized ZnO-NPs possess best antimicrobial action under visible light. The results illustrated in Fig. 3a–f. Panels a–c show the growth curves obtained from the readings of OD, till 8 h of incubation, at selected ZnO concentrations. While panels d–f show the inhibition viability, with ZnO-NPs of very small size (∼12 nm), that inhibited about 95 % of the growth with respect to the control (0 mM). Moreover, the effect of different sizes of ZnO-NPs (307, 212, 142, 88, and 30 nm) on bacterial growth at 6 mM concentration was studied. By analyzing growth curves at OD_600 nm_, we could perform colony count, and obtain the growth inhibition percentage which was plotted against particle sizes (Fig. 3g). The number of viable cells recovered, at a certain point of growth, showed significant decrease with decreasing ZnO-NPs particle size (Fig. 3h). This was attributed to the increased reactivity of small size NPs, as the generated amount of H_2_O_2_ is significantly dependent on the surface area of ZnO-NPs [12]. On the other hand, the antibacterial activity depends on the concentration and the crystalline structure of ZnO. Increased cell death achieved by increasing ZnO concentrations, which disrupt mitochondrial function, stimulating lactate dehydrogenase leakage and changing the morphology of the cell at concentrations of 50–100 mg L^−1^ [90]. Yamamoto [51] found that the higher concentration and the larger surface area can obtain the better antibacterial activity. Also, Jones et al. [8] fulfilled their antibacterial tests using four types of NPs (TiO_2_, MgO, CuO, and CeO_2_) which have not considerably inhibited the bacteria growth (more than 10 mM). These results contradicted those obtained for ZnO and Al_2_O_3_ NPs, which exhibited significant growth inhibition. The researchers found that just 2 mM of ZnO-NPs with reduced sizes decreased bacterial growth by 99 %. This result confirmed that the antibacterial activity also depended on the size, and it was probably due to the internalization and subsequent accumulation of NPs inside the cells until the particles reached the cytoplasmic region. In addition, the authors performed the tests in dark, and observed that the antimicrobial effect was weaker. Thus, they concluded that only an ambient laboratory environment could achieve the optimum bactericidal effects, and the reduced size and concentration enhanced this effect. The different conditions, including the type of culture medium and the bacterial cells number, contribute to the variations in antibacterial activity results. Overall, this flourishing study confirms that ZnO-NPs are successful candidates for further antibacterial applications owing to their extensive growth inhibition, as a result of the low concentration and smaller particle size. A related study on five oral bacteria showed that ZnO has bacteriostatic effects on two bacterial strains: *Lactobacillus salivarius* and *Streptococcus sobrinus*. However, few inhibitions were observed on the three other types (*P. aeruginosa*, *Streptococcus mutans*, and *S. aureus*) according to those tests conditions [90]. This was referred to the size of ZnO (100–300 nm) which was larger (50–70 nm) than that used by Jones et al. In a similar manner, Zhang et al. [62] performed an interesting study using ZnO nanofluids. Their results showed bacteriostatic action towards *E. coli*, which increased at higher ZnO-NPs concentrations and reduced size. Furthermore, the authors used SEM analyses to examine the morphological changes. The data showed that ZnO-NPs interacted with *E. coli* membrane wall resulting in considerable wall damages, which in turn collapsed the cell membrane. Similarly, increasing the concentration of the ZnO dispersions to 10 mM with extended exposure time (30 min) has inhibited totally *E. coli* growth [36]. A parallel study was made by Jalal et al. [9] who obtained strong antibacterial activity against *E. coli* at increased concentration. As a result, an increase in H_2_O_2_ amount was produced from ZnO surface, a lethal species to bacteria.

**Fig. 4:**
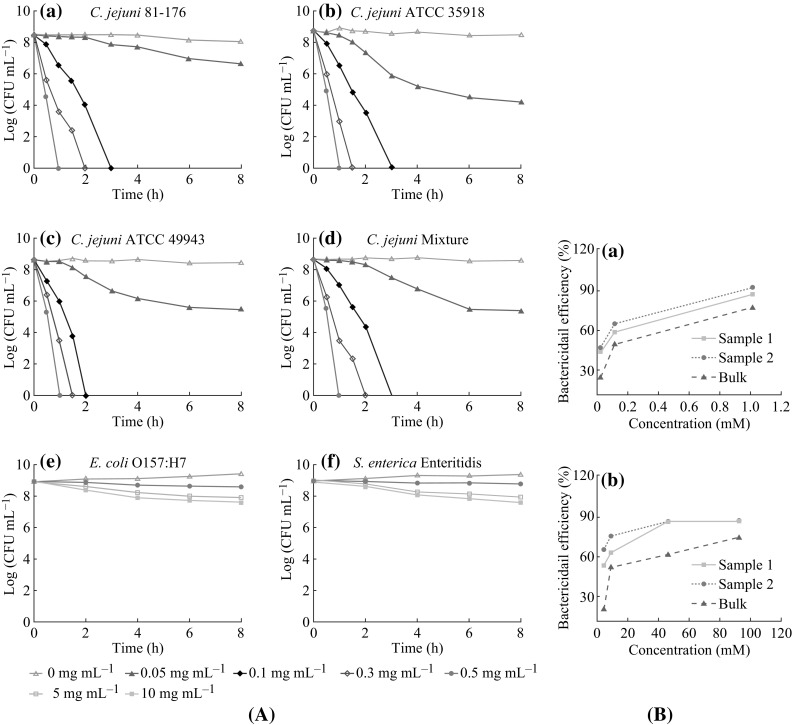
**A** Bactericidal efficacies of ZnO suspensions, for tested samples namely *sample 1*, *sample 2*, and *bulk* with three different particle sizes after 24 h incubation. Reproduced by permission from Padmavathy and Vijayaraghavan [12]. **B** Antibacterial activity of ZnO-NPs towards: *Enteritidis* and *E. coli* O157:H7, adapted from Xie et al. [91]

Also concentration-dependent bactericidal activity of ZnO-NPs was fruitfully evaluated by Xie et al. [91] toward four foodborne pathogens (*C. jejuni* is known as the most common foodborne pathogen, *Salmonella enterica Enteritidis*, *E. coli* O157:H7, and *Salmonella* strains). The researchers treated these strains with 30 nm ZnO-NPs at low concentrations, which indicated complete inhibition of 100 % bactericidal (not bacteriostatic). The ZnO concentrations were 0, 0.025, 0.03, 0.04, 0.05, and 0.10 mg mL^−1^. *C. jejuni* cells were killed at 108 CFU mL^−1^ after only 3 h, at selected concentrations of 0.1, 0.3, and 0.5 mg mL^−1^. This result confirms the potent antibacterial effect of ZnO-NPs toward this particular bacterial species at much reduced concentrations, and this finding is highly beneficial in food packaging. Also, they revealed that three strains of this bacterium possess greater degree of susceptibility toward ZnO-NPs, as determined from the MIC (Fig. 4A: a–d), and considered a lethal effect. By contrast, *S. enterica serovar Enteritidis* and *E. coli* O157:H7 showed a reduction in viable cells number after 8 h exposure, and the growth inhibition was determined by counting the numbers of CFUs for the bacteria (Fig. 4A: e, f). In case of *E. coli* O157:H7 (a major foodborne pathogen) showed 100 % growth inhibition in the presence of a ZnO concentration which is approximately 8–32 times that used for *C. jejuni*. The efficiency of ZnO-NPs against *Salmonella* was also tested at lower concentration. ZnO-NPs concentrations that are 20–100 times were required for decreasing 1–2 logs of cells viability. The result showed that ZnO damaged the membrane integrity. Moreover, the antibacterial activity toward *S. enterica serovar Enteritidis* was also determined at lower concentration. Recently, Palanikumar et al. [92] reported that ZnO-NPs inhibit the growth of *Staphylococcus epidermidis* in a size- and concentration-dependent manner. Their findings revealed as well wide spectrum of antimicrobial activities of ZnO-NPs against various microorganisms (Table 2).

**Table 2:** Selected studies show concentration-dependent antibacterial activity of ZnO-NPs

Studied concentration	Tested organism	ZnO synthesis	References
0–10 mM	*S. aureus* and *E. coli*	Hydrolysis-zinc acetate	Reddy et al. [50]
0.125, 0.25, 0.5 g dm^−3^	*E. coli*	Microwave-zinc acetate decomposition	Jalal et al. [9]
0.1, 0.3, and 0.5 mg mL^−1^	*C. jejuni*, *E. coli* O157:H7, *S. enterica serovar Enteritidis* *Salmonella*	Suspensions in ddH_2_O	Xie et al. [91]
20, 50, 100 μL	*Pseudomonas aeruginosa* and *E. coli*	Wet chemical method	Chitra and Annadurai [139]

### Minimum Inhibitory Concentration (MIC)

For discussing MIC and MBC measurements, a comparison between a study reported by Emami-Karvani and Chehrazi [11] and Reddy et al. study [50] was presented. Briefly, an agar diffusion test was performed by inoculating the targeted bacteria (*E. coli* and *S. aureus*) on NA using ZnO-NPs at a defined concentration range. The CFU for each plate was then counted and incubated for 24 h to determine the bacteria growth rates. Autoclaved distilled water (0.1 mL) was added to the plates that did not show any bacterial growth. The culture was then transferred to a fresh medium without ZnO-NPs. MBC is determined as the lowest concentration that showed no bacteria growth in the fresh medium, whereas MIC is the lowest NP concentration at which colonies are observed on the surface of the fresh medium. In other words, MIC is the concentration which impedes and absolutely prevents bacterial growth. It was found to be 1.5 and 3.1 mg mL^−1^ for *S. aureus* and *E. coli*, respectively. These results were consistent with those obtained by Reddy et al., which was 1 mg mL^−1^ for *S. aureus* and 3.4 mg mL^−1^ for *E. coli*. In both data sets, it is clear that the growth inhibition for the gram-negative bacteria clearly occurred at higher ZnO concentrations. This finding confirms that gram-positive bacteria are more susceptible to inhibition compared to gram-negative bacteria. The inhibition accounts for variations in cell physiology, cell wall constitution, and the metabolism [93, 94]. Additionally, Xie et al. [91] reported that the MIC of ZnO-NPs (30 nm) toward *C. jejuni* (0.05–0.25 mg mL^−1^) was 8–16-fold lesser than *E. coli* O157:H7 and *S. enterica serovar Enteritidis* (0.4 mg mL^−1^). It is also obvious that ZnO-NPs activity was concentration dependent.

MIC was determined recently in a different approach by Salem et al. [95] against two pathogens enterotoxic *E. coli* and *Vibrio cholerae*, causative agents of diarrheal diseases leading to death. The researchers introduced growth curve test besides INT reduction assay in 96-well plates supplemented with bacterial culture and ZnO-NPs and Ag-NPs. The OD (600 nm) was measured after 16 h incubation at 37 °C. MIC was determined by the lowest concentration of each NPs, which inhibited the growth. Bacterial growth was defined by an at least 2-fold increase of the OD_600 nm_ with respect to the negative control (growth medium only). Additionally, INT assay (a tetrazolium reduction assay) was performed using *P*-iodonitrotetrazolium violet INT to determine lack of metabolic activity and reveal the growth inhibition. INT was added to the cultures in 96-plates and incubated 30 min until a color change occurred. MIC was defined as the lowest NPs concentration which did not show any color change. A higher efficacy was exhibited by ZnO-NPs compared to Ag-NPs. ZnO-NPs concentration of 1.6 × 10^5^–1.2 × 10^6^ mL^−1^ was necessary for killing both pathogens, while Ag-NPs concentration of 5 × 10^6^–1.2 × 10^7^ mL^−1^ was needed to kill the pathogens.

### Surface Defects

Other factors that play vital roles on the mechanism are surface defects and surface charges as the surfaces of ZnO-NPs containing numerous edges and corners, and thus have potential reactive surface sites. In spite of its simple chemical formula, ZnO has very rich defect chemistry [96], which is associated with its antimicrobial activity. Surface defects strongly affect the toxicity of ZnO. For instance, Padmavathy and Vijayaraghavan [12] suggested that the antibacterial action of ZnO-NPs is due to the membrane injury caused by defects such as edges and corners, which results from the abrasive surface of ZnO. Interesting applications of ZnO-NPs can be achieved by controlling the defects, impurities, and the associated charge carriers. Defects extensively change grain boundary properties and the IV characteristics [23].

Finally, Wang et al. [97] proposed the orientation of ZnO which affects the biocidal activity of ZnO because of its various randomly oriented spatial configurations, which exhibits higher antibacterial action compared with those of regularly arranged structures [12, 80]. As well, Ramani et al. [79] referred the toxicity of nanostructured ZnO to their orientation, while, it has been found as irrelevant for crystallographic orientation [98]. The inconsistency made the effect under research.

Besides ZnO, other NPs including Ag, MgO, TiO_2_, CuO, CaO, CeO_2_, SiO_2_, Al_2_O_3_, and Fe_3_O_4_, exhibit antimicrobial properties and are safe to humans and animals [99]. Metal NPs are known for their extremely ionic characteristics and are synthesized with different morphologies that exhibit remarkable crystallinity and highly surface area. The surfaces of these NPs are reactive due to plentiful corners and edges [100]. However, some metal oxides are highly toxic, for example, Al_2_O_3_ toxicity toward many cells [8]. Moreover, Ag-NPs toxicity was reported [3, 101, 102] and TiO_2_ was revealed to kill a number of bacteria [8, 103, 104]. Adding such metals to ZnO as precursor could lead to remarkable results, and the precipitation method reported by Zhang [68] who reported that silver-loaded ZnO showed extreme increase of ZnO antibacterial activity. Ag-loaded ZnO speculated as a new kind of precursor for inorganic antibacterial agents.

## Proposed Mechanisms of Antibacterial Activity

### Reactive Oxygen Species (ROS) Generation

The toxicity of ROS to bacteria is attributed to their high reactivity and oxidizing property [105], it has been reported that aquatic ZnO-NPs suspensions produce augmented level of ROS. Numerous studies have considered ROS generation as the major cause of nanotoxicity [7, 56, 105–107]. The photocatalytic generation of ROS has been a major contributor to the antibacterial activities of various metal oxides [108]. Several studies indicated ROS formation as the main mechanism responsible for ZnO-NPs antibacterial activity [9, 12, 51, 60, 62]. Raghupathi et al. [13] showed that enhanced ZnO antibacterial activity was due to the increased ROS production from ZnO under UV exposure. Such reactive species are superoxide anion (O^2^), hydrogen peroxide (H_2_O_2_), and hydroxide (OH^−^). The toxicity of these species involves the destruction of cellular components such as lipids, DNA, and proteins, as a result of their internalization into the bacteria cell membrane. However, the role of ROS in the antimicrobial actions has become an argument issue among the researchers in this field [13]. The creation of ROS seems to be contradictory since a number of studies have revealed this mechanism under light exposure, as mentioned earlier. While alternative studies reported the activity even in the dark [56, 74]. The creation of ROS in the dark was observed by Hirota et al. [74] by testing ZnO-NPs toward *E. coli*. They found that the activity can occur under darkness, producing superoxide species; which is in consistence with Jones et al. findings [8]. Such consistent results give a sign of possibly further mechanisms so far to be determined to produce reactive species without illumination and in dark. Therefore, further studies are required to explain these findings deeply. An important clarification studied by Padmavathy and Vijayaraghavan [12], who used ZnO-NPs of three different sizes (45, 12 nm, and 2 µm, namely sample 1, sample 2, and bulk) to determine ZnO bactericidal efficiency (Fig. 4B). They found that the smaller sized, 12 nm showed best efficiency compared to 45 nm and 2 µm. This was attributed to ROS release on ZnO-NPs surface under both UV and visible light, and the ROS release caused lethal bacterial injury. The researchers explained the production of ROS (OH^−^, H_2_O_2_, and ${\text{O}}_{2}^{2 - }$) on ZnO surface and proposed a correlation between photon reactions and the antibacterial activity as follows.

The electron and hole interacts with water (H_2_O) to produce ^•^OH and H^**+**^. In addition, O_2_ molecules (suspended within the mixture of bacteria and ZnO) yield superoxide anion (${}^{ \bullet }{\text{O}}_{2}^{ - }$), which reacts with H^**+**^ to produce ${\text{HO}}_{2}^{ \bullet } .$ Afterward, ${\text{HO}}_{2}^{ \bullet }$ interferes with electrons generating hydrogen peroxide (^•^HO_**2**_); which combines with H^**+**^ giving hydrogen peroxide (H_2_O_2_) molecules. The latter are capable to enter the membrane where they either damage or kill the bacteria. H_2_O_2_ generation mainly relies on the surface of ZnO-NPs to yield additional active molecules. There is a linear proportionality between the concentrations of H_2_O_2_ produced in ZnO slurry and the ZnO particle size [88]. The mentioned researchers expressed the generated ROS by chemical equations as follows:

4 $${\text{ZnO}} + {\text{h}}\upnu \to {\text{e}}^{ - } + {\text{h}}^{ + } ,$$

5 $${\text{h}}^{ + } + {\text{H}}_{2} {\text{O}} \to {}^{ \bullet }{\text{OH}} + {\text{H}}^{ + } ,$$

6 $${\text{e}}^{ - } + {\text{O}}_{2} \to {}^{ \bullet }{\text{O}}_{2}^{ - } ,$$

7 $${}^{ \bullet }{\text{O}}_{2} + {\text{H}}^{ + } \to {\text{HO}}_{2}^{ \bullet } ,$$

8 $${\text{HO}}_{2}^{ \bullet } + {\text{H}}^{ + } + {\text{e}}^{ - } \to {\text{H}}_{2} {\text{O}}_{2} .$$

The superoxides and hydroxyl radicals cannot penetrate into the membrane due to their negative charges [91]. Thus, these species are found on the outer surface of the bacteria, by contrast, H_2_O_2_ molecules are able to pass through the bacterial cell wall, subsequently leading to injuries and destroy, and finally triggering cell death [40, 88]. When ZnO-NP kills or interacts with the cell membrane, the particles most probably stay firmly adsorbed at the surface of the left over/killed bacteria blocking additional antibacterial activity. Once ZnO-NPs are in the growth media, they will carry on releasing peroxides covering the entire surfaces of the dead bacteria. Therefore, this continuous peroxide release leads to higher bactericidal efficacy.

The production of ROS has been concerned in the onset and development of many diseases (such as cancer, atherosclerosis, diabetes, and neurodegeneration). In this regard, investigation of sensitive ROS detection probes has been of utmost importance and was carried by some researcher. A fluorescent dye dichlorodihydrofluorescein diacetate (DCFH-DA) has been used recently to detect the intracellular ROS levels in bacteria and cancer cell lines [109, 110]. Thus, the mechanism related to the formation of ROS in bacteria and cell line was clearly examined by the measurement of ROS level using DCFH-DA [111]. Production of ROS by metal NPs in cell lines has been revealed by several studies [111–113], and referred to the inhibition of the respiratory enzymes [114]. Previously, it was documented that ROS production in bacteria was mostly due to the autoxidation of NADH dehydrogenase II in the respiratory system [115].

The mechanism of antibacterial activity by ROS generation due to treatment with ZnO-NPs using DCFH-DA dye was studied by Dwivedi et al. [109]. The dye passively enters into the bacterial cell, and then is hydrolyzed by cell esterases to DCFH which is oxidized to DCF, a highly fluorescent compound dichlorofluorescein in the presence of ROS. The fluorescence was determined by flow cytometry or microplate reader, the fluorescent intensity is proportional to the amount of ROS [116].

$${\text{DCFH-DA}}\mathop{\longrightarrow}\limits[{\text{esterases}}]{}{\text{DCFH}} \to {\text{DCF}} .$$

### Zinc Ions (Zn^2+^) Release

One of the main proposed antibacterial mechanisms for ZnO-NPs is release of zinc ions in medium containing ZnO-NPs and bacteria [47, 59, 110, 117, 118]. The released Zn^2+^ has significant effect in the active transport inhibition as well as in the amino acid metabolism and enzyme system disruption. Several studies have believed that the leaked Zn^2+^ into growth media responsible for ZnO nanotoxicity and the dissolution of ZnO-NPs into Zn^2+^ were found as size dependent. Therefore, engineered nanostructures might modify their toxicity by manipulating the dissolution rate [80, 107, 117, 119, 120]. Kasemets et al. [57] have shown that the release of Zn^2+^ ions was a logical cause of ZnO toxicity toward *Saccharomyces cerevisiae* bacteria (these bacteria is highly considered in food processing). According to this hypothesis, ZnO-NPs toxicity is referred to the solubility of Zn^2+^ in the medium including the bacteria. Therefore, solubilized low concentrations Zn^2+^ can induce a comparatively high tolerance in bacteria. Reddy et al. [50] treated *E. coli* with a low concentration (1 mM) of solubilized Zn^2+^. On contrast, Sawai [48] and Jiang et al. [121] described the contribution of Zn^2+^ to the antimicrobial efficacy of ZnO-NPs as minor due to the low concentrations of solubilized Zn species released from ZnO dissolution. The aforementioned studies reported the mechanism as predominant. So the dissolution phenomenon is somewhat under debate, although it has been adopted and accepted. Additionally, Zn^2+^ release would be limited by an inherent ZnO property, mentioned earlier ZnO stability in water. The insolubility of ZnO impedes the distribution of zinc ions into the medium and thus limits this antimicrobial effect [118], unless ZnO capped or stabilized. The physicochemical properties of ZnO-NPs and dissolved zinc species depend upon the medium components. Though, Pasquet et al. [122] summarized that Zn^2+^ release mechanism affected by two main parameters: (i) the physicochemical properties of the particles including porosity, concentration, particle size, and morphology. (ii) The chemistry of the media: the pH, UV illumination, exposure time, and existence of other elements. However, the influence of these parameters is not entirely elucidated. Peng et al. [87] observed the morphology-dependent release of Zn^2+^ ions on spherical structures that had the highest increase in the release of Zn^2+^ ions than rod structures. It was elucidated on the fact that smaller surface curvature of sphere causes high equilibrium solubility. Also, Wang et al. [123] studied the morphology-dependent dissolution of metal ions. Leung et al. [85] proposed that the most probable mechanisms can be influenced by surface modifications because both the liberation of Zn^2+^ ions and ROS creation occur on NPs surface. Moreover, the surface properties affect the reactions on the bacterial cell walls. In this regards, characterizations techniques assist in recognizing the mechanisms such as SEM, XRD, TEM, and ESI. For example, ESI is used to investigate the elemental distribution of ZnO particle surfaces. In our study [83], where ESI elemental mapping results showed a higher O:Zn ratio on the surface of ZnO-rod structure but lower O:Zn ratio on ZnO-plates surfaces. Therefore, ZnO-rod tends to have relatively higher O:Zn ratio than other ZnO-structures, i.e., higher amounts of oxygen atoms on rod surface, which generates ROS causing intense oxidative stress toward the bacteria. Currently, it was found that Zn^2+^ of ZnO is capable to interact with protein and has a potential effect to HSV-1 pathogenesis [109].

### Different Probable Mechanisms

**Fig. 5:**
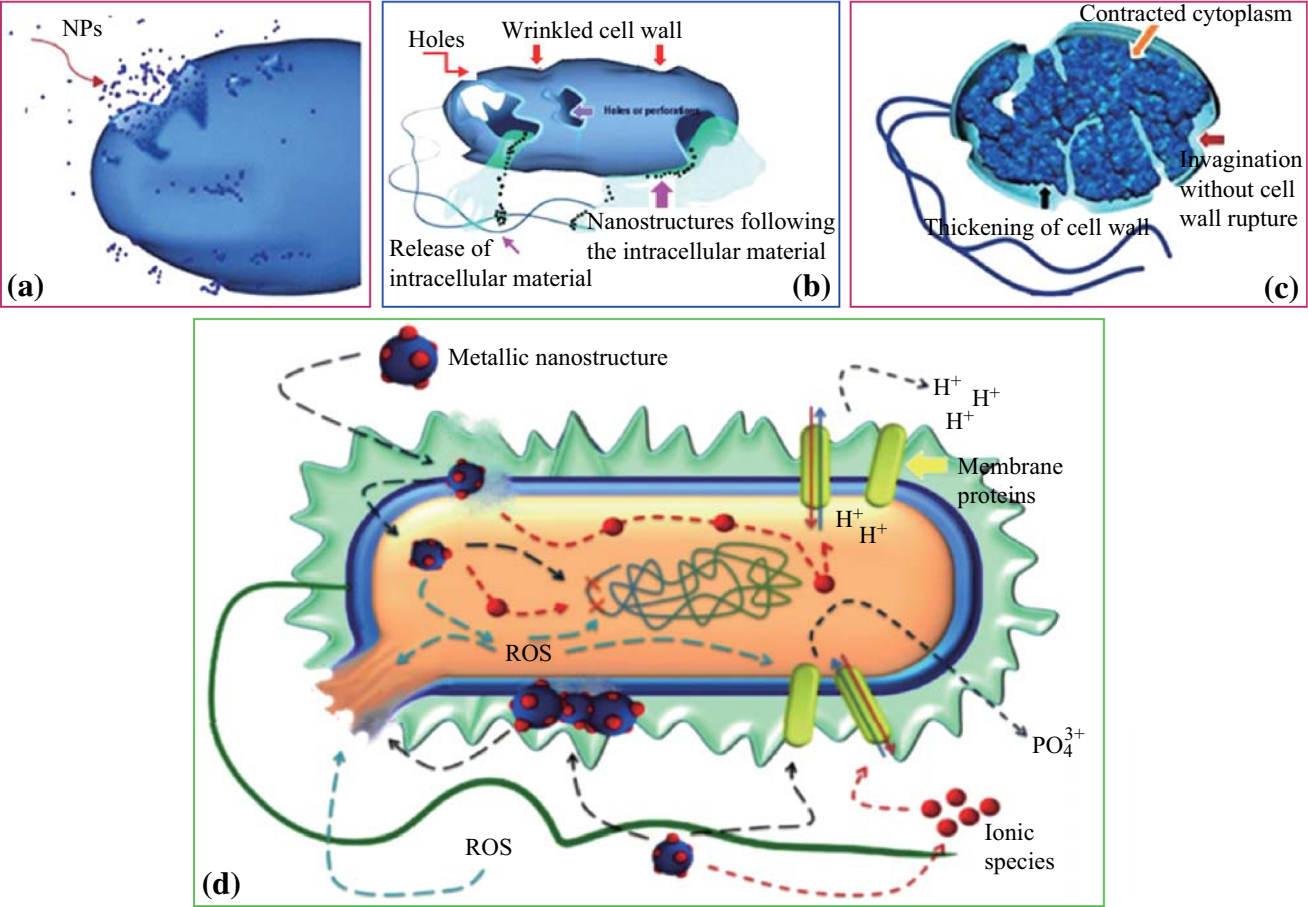
**a** NPs internalization into the cell and translocation. NPs penetrate through holes, pits or protrusions in the cell wall. **b** Schematic representation of collapsed cell showing disruption of cell wall and extrusion of cytoplasmic contents. **c** Bacterial cell showing important variations in envelope composition (slight invaginations and thickening of cell wall) and extrusion of cytoplasm. **d** Probable mechanisms, involves the following: metal ions uptake into cells, intracellular depletion, and disruption of DNA replication, releasing metallic ions and ROS generation and accumulation and dissolution of NPs in the bacterial membrane. Reused from Díaz-Visurraga et al. [128]

Further suggestions for the bactericide achievement are the inhibition of energy metabolism, once NPs have internalized bacteria. ZnO-NPs are bactericidal and thus disrupt membrane causing membrane dysfunction, resulting in their internalization into the bacteria (Fig. 5a). ZnO internalization is controlled by the particle size, surface chemistry, defects, and functionalization. Compared with cells exposed to Ag-NPs and ions, the cell energy is reduced due to the decline in the adenosine triphosphate levels and the essential energy molecule, and destabilization of the outer membrane was followed [4]. The effect of the pH value of the reaction medium in the antibacterial activity mechanism has been considered. A value in the neutral region (pH 7.5) was assumed to have no effect on the antibacterial activity in absence of light [124]. Meanwhile Sawai et al. [60] found that the activity could not be detected for pH in range of 5.5–8.0. While Stanković et al. [125] varied the pH value of the starting reaction solution from 8 to 12 that enabled in changing the morphology from micro-rods to nano-spheres, resulting in an efficient bacteriostatic activity.

There is a strong trend that considers two mechanisms underlying the interaction of NPs with bacteria, to be mainly concerned [13]: (i) excessive ROS generation, mostly hydroxyl radicals (HO^•^) and singlet oxygen (^1^O_2_) [60, 80, 126, 127], and (ii) NPs precipitation on the bacterial exterior; or NPs gather in the cytoplasmic area or in the periplasm space, thus, disrupt the cellular activities, resulting in membranes disturbance and disorder [7, 40]. In this way, Zhang et al. [62] referred some of the effect to a direct liaison between NPs and the membrane as well as to ROS generation nearby bacteria membrane. Zhang et al. [62] suggested a creation of electrostatic forces when *E. coli* treated with ZnO. Stoimenov et al. [100] also proposed this electrostatic interaction between NPs and bacteria cell surface as a cause of growth inhibition, and that the total bacterial charge is negative, because of the excessive formation of separated carboxyl groups. Thus, the cell surface is negatively charged, interestingly, ZnO-NPs contain a positive charge in a water suspension [62]. Such reverse charges enhance the total effect by creating electrostatic forces, which serve as a powerful bond between NPs and bacterial surface. As a result, the cell membrane is damaged. Additionally, Brayner et al. [7] observed that the interaction between *E. coli* and ZnO-NPs yields cell wall disorganization followed by internalization of NPs into the cells. They recognized a substantial damage to *E. coli* with disorganized cell walls by SEM images which showed the changed morphology, a consequence of intracellular content leakage. Also, the images showed ZnO-NPs both inside and outside the cell bordered probably by lipopolysaccharides released of bacteria. They explained the capability of ZnO-NPs to reduce the bacteria growth. It was attributed to membrane disruption and raises its permeability, which in turn causes the gathering of ZnO-NPs inside the membrane and then reaches the cytoplasm. These results are similar to those obtained by Díaz-Visurraga et al. [128] shown in Fig. 5a–c. An identical finding showed the attachment of ZnO-NPs to outer cell wall and passing in the inner wall, causing disruption of the membrane and consequent disorder and leakage [46]. In a similar manner, Xie et al. [91] found that the action of ZnO-NPs on *C. jejuni* has stimulated morphology disorder, intracellular components outflow, and considerable release in gene expression of oxidative stress of *C. jejuni*. In addition to the aforementioned mechanisms, ZnO-NPs have abrasive surface texture which influences the antibacterial mechanism, which in sequence destroys the bacterial membrane [12]. The abrasive property of ZnO has been recognized and referred to its surface defects [100]. These defects such as corners, edges, and chemistry defects have a major impact on the antibacterial activity in the mechanical damage on cell wall. Surface defects play an important role; Ramani et al. [129] reported that ZnO nanostructures antibacterial activity is surface-dependent defect which in sequence are shape dependent. Although the detailed mechanism of ZnO antibacterial activity is under discussion, a three most widely accepted, and reported hypothetical mechanisms in the literature [128] are: (i) metal ions uptake (translocation and particle internalization) into cells followed by depletion of intracellular ATP production and disruption of DNA replication [130], (ii) ROS generation from NPs metal oxides and ions with subsequent oxidative damage to cellular structures [131], and (iii) changes in bacterial membrane permeability (progressive release of lipopolysaccharides, membrane proteins, and intracellular factors) and dissipation of the proton motive force as a result of accumulation and dissolution of NPs in the membrane [132]. These mechanisms were illustrated in Fig. 5a, b, d along with other predicted ones previously mentioned.

## A Study of ZnO-NPs Antibacterial Response to *E. coli*

We present here briefly one study in which we used ZnO-NPs (80 nm) produced via French process [28] of high purity (>99.97 %). A stock solution was prepared in ddH_2_O, and vigorously vortexed (3 min) and subjected to high ultrasonication (30 min) prior to addition to culture mixture, and diluted to concentrations 1–4 mM. Two samples were used to treat *E. coli* (ATCC 25922), ZnO-AP (as purchased), and ZnO–O_2_ (oxygen annealed). ZnO powder was annealed at 700 °C in an annealing tube furnace (model Lenton) under oxygen ambient for 1 h. The gas flow was regulated at 2.4 L min^−1^. Bacteria and ZnO mixture were prepared in 96-well plate including control and incubating in 5 %, CO_2_, 37 °C incubator chamber, as described below.

Bacterial culture conditions for the antibacterial tests were conducted. *E. coli* was freshly prepared, grown in NA broth, and incubated for 24 h at 37 °C, and then supplemented with four concentrations of ZnO-NPs suspensions to study the bacterial growth rate. Then the inoculum preparation and inoculation was carried by preparing standardized inoculums of *E. coli* containing approximately 5 × 10^5^ CFU mL^−1^. This was accomplished by diluting the 0.5 McFarland suspension 1:150, resulting in a tube containing approximately 1 × 10^6^ CFU mL^−1^. Within 15 min after the inoculum has been standardized as described above, a 150 µL of the adjusted inoculums was added into the 96-well microplate containing 150 µL ZnO in the dilution series, and triplicated for each concentration. The controls, positive control (ZnO and TSB), and negative control (bacteria and TSB) were prepared for each set of experiment. All types of serial concentration of ZnO were mixed with relevant bacteria. This resulted in a 1:2 dilution of each ZnO concentration and a 1:2 dilution of the inoculums 5 × 10^5^ CFU mL^−1^.

Then the mixture in the 96-well plate were exposed to UVA light (390 nm, 1.8 mW cm^−2^) for 20 min, and consequently OD measurements were held hourly up to 8 h, and then after 24 h to determine the percentage growth inhibition. Additionally, cells were fixed to be viewed by FESEM to observe the bacterial morphological changes after treated with ZnO-NPs. Cells were mounted on specimen stub using a double-sided carbon tape and coated with platinum.

The percentage inhibition (after 24 h incubation) was calculated from the OD readings as follows.

**Fig. 6:**
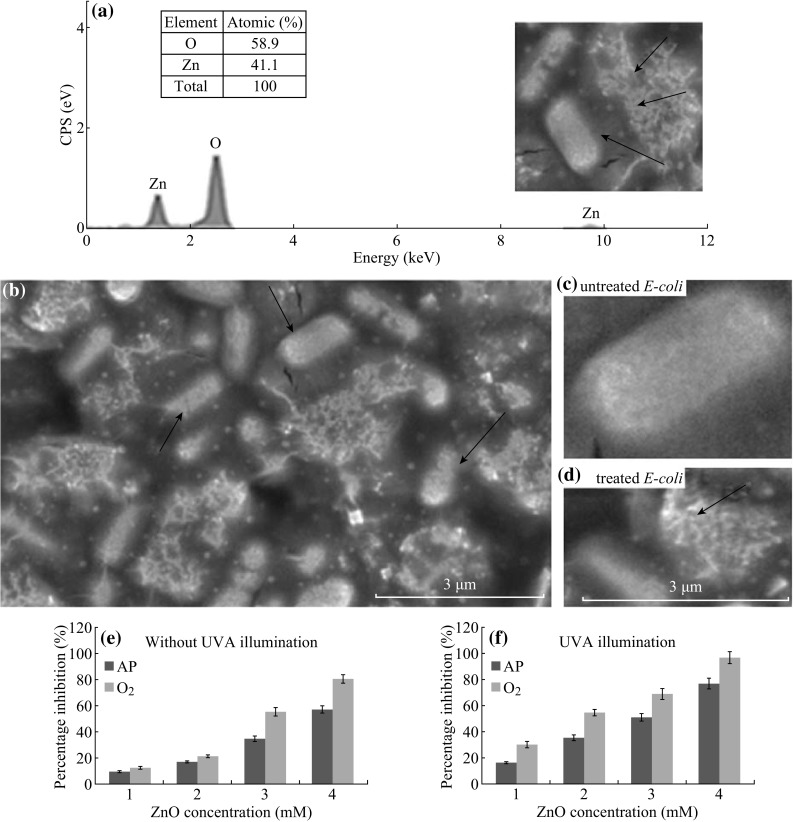
**a** EDS spectrum of *E. coli* in ZnO mixture, **b** FESEM micrographs of *E. coli* exposed to ZnO, *arrows* show ZnO particles on the bacteria surface, **c** untreated bacteria cells, **d** *E. coli* treated with ZnO, and **e**, **f** percentage inhibition of *E. coli* treated with ZnO-AP and ZnO–O_2_ at different concentrations with and without UVA illumination, respectively (experiment was done by authors of this manuscript, triplicated)

Results were displayed in Fig. 6, it was observed that higher concentration of ZnO caused higher bacterial inhibition. Moreover, the UV illumination increased the percentage inhibition of bacteria (Fig. 6e, f). As it is known that ZnO absorbs UV light, it has an excellent photocatalytic property, thus it is believed that UV stimulated the ZnO in the mixture to release electrons, which leads to produce more oxygen within the mixture. This phenomenon induced the generation of ROS from ZnO surface, as described earlier. ZnO–O_2_ had exhibited the highest growth inhibition capability toward *E. coli* than ZnO-AP. This was most likely attributed to the higher surface defects induced by annealing. The oxygen annealing stimulated a high level of oxygen atoms to be absorbed onto the surface of ZnO, which was revealed by the EDS spectrum (Fig. 6a). Additionally, the release of electrons was believed to interact with the bacteria where the electron discharges the bacteria membrane halting the bacterial growth.

The FESEM images (Fig. 6b–d) show ZnO-NPs on the bacteria surfaces, which probably inhibited the growth due to the generated ROS into the mixture of ZnO with *E. coli*, which was enhanced by the UV exposure and also the annealing process causing oxygen absorption on the surface of ZnO samples. The high amount of oxygen on the surface induced ROS release in ZnO suspension. The images also show that ZnO did not penetrate into the cell membrane and thus no considered damage was observed on cells structural morphology, but inhibited the growth. This might be probably suggested that the size of the particles did not allow their penetration into the cell wall and membrane. Our results of this experiment were in agreement with some studies [9, 12, 13], which showed that enhanced ZnO antibacterial response is referred to the increased ROS production under UV light. And also with Zhang et al. [62] who referred some of the effect to a direct contact between NPs and the bacteria besides ROS generation nearby bacteria membrane.

## ZnO-NP as an Antibacterial Agent in Food

Research on ZnO-NPs as antibacterial agent has become interdisciplinary linking physicists, biologists, chemists, and medicine, hence it is the wide spread of their applications. One of these essential applications is in food industry; as an antibacterial agent in food packaging and towards foodborne pathogen. Nanomaterials possess great concern in food technology for their high reactivity, enhanced bioavailability and bioactivity, and have creative surface possessions [133]. Some of the main benefits of using NPs in food nanotechnology are the addition of NPs onto food surfaces to inhibit bacterial growth, also using of NPs as intelligent packaging materials and for nano-sensing [134]. Among these NPs, ZnO-NPs developed as a successful candidate in the food industry [71, 135]. The antibacterial influence of ZnO-NPs against foodborne pathogens stimulates proficient applications in food packaging, and can be introduced in food nanotechnology. Duncan [136] reported about recent applications of antimicrobial NPs on food, to achieve high barrier packaging materials, and nano-sensors using NPs to trace food-relevant analytes such as foodborne pathogens. Generally, food industry witnessed revolution by the implementation of nanotechnology.

### Food Pathogens

Recently, the need for novel technologies to control foodborne pathogens is increasing, due to the alarming increase in fatalities and hospitalization worldwide. Foodborne illnesses are an increasing major health problem in both developing and developed countries. Each year, 1 million people in the UK acquire foodborne illness, 20,000 people undergo hospitalization, and 500 deaths. As announced by the* Food Standards Agency*, which is a program for the reduction of food-borne diseases in the UK. The spread of foodborne diseases can result in many social problems such as poverty, health problems, and even economic issues. Moreover, in recent years, the pathogenic bacteria have exhibited antimicrobial resistance, and this emerged as hot subject of discussion among researchers in this field. Some most common bacterial foodborne pathogens are *C. jejuni*, *C. perfringens* (is the cafeteria germ), *Salmonella* spp., and *E. coli* O157:H7. Several studies were conducted to determine the interaction of ZnO-NPs with foodborne pathogens, since ZnO-NPs are listed as being safe (US FDA). Studies showed that ZnO-NPs can inhibit and kill common as well as major foodborne pathogens. The bactericidal activity of ZnO-NPs (8–10 nm size) against *E. coli* DH5α and *S. aureus* was examined and found to be effective at 80 and 100 µg mL^−1^. These concentrations disrupted the cell membrane causing cytoplasmic leakage [137]. Narayanan et al. [138] tested the antibacterial activity of ZnO-NPs against some human pathogens such as *P. aeruginosa*, *E. coli*, *S. aureus*, and *E. faecalis*. They emerged with the result that ZnO-NPs have strong antibacterial activity toward these human pathogens. Likewise, the antimicrobial activity of ZnO-NPs was studied [139] toward *P. aeruginosa* and *E. coli* which were isolated from mint leaf extract and frozen ice cream, and ZnO was prepared using wet chemical method, yielding spherical morphology with smooth surface, of concentrations 20, 50, and 100 μL. Both bacteria showed decreased growth rate at the highest concentration 100 μL, and they explained the growth inhibition as a result of cell membrane damage through penetration of ZnO-NPs. They concluded that ZnO-NPs synthesized by wet chemical method are potential antibacterial agents in food preservation and packaging.

### Food Packaging Applications

Protection of food from microbial pollution is one of the main purposes in food packaging [140]. The emergence of nanotechnology assisted to present novel food packaging materials with antimicrobial properties and with novel nano-sensors to trace and monitor the food [135]. Several studies have addressed the antibacterial properties and potential applications of ZnO-NPs in food processing. For example, ZnO has been included into a number of food linings in packaging to avoid spoilage plus it maintains colors. ZnO-NPs provide antimicrobial activity for food packaging. Once they are introduced in a polymeric matrix, it permits interaction of food with the packaging possessing functional part in the conservation. Other benefits also are achieved such as the barrier properties, constancy, and mechanical capability [71]. The use of polymer nanotechnology in packaging was introduced by Silvestre et al. [135] to achieve novel way of packaging that mainly meet the requirements of protection against bacteria. These new materials with improved antimicrobial properties permit also tracking of food during storage and transfer.

#### Active Packaging

Recently, active food packaging systems as an emergent approach in food packaging have replaced the conventional packaging systems to achieve effective performance. The conventional approach uses a passive barrier to protect food against the surrounding atmosphere [141]. While, active packaging create an effective antimicrobial action on food, and saves the inert products from the environmental factors. The liberation of the NPs, which acts as bacteriostatic or bactericidal agents onto the food surface where bacteria reside, halts the growth and thus prevents food from spoilage [141]. This type of active packaging is also called *antimicrobial packaging*, where direct interaction occurs between the product and the NPs leading to the killing or inhibition of bacterial growth on food surfaces [142]. Accordingly, direct addition of highly concentrated antibacterial to a packed food is not recommended. The inclusion of antibacterial agents assists either bacteriostatic or bactericidal materials to gradually diffuse into the food matrix. Hence, reducing the possibility of pathogen contamination and thus a safe product with an extended shelf life was obtained. Ahvenainen [143] stated that active packaging satisfies the consumer demand as it enhances safety, with more natural products of extended life time. The packaging materials are firstly characterized before incorporating with ZnO-NPs by microscopic and spectroscopic techniques. In XRD, a scattered intensity of X-ray beam on the sample provides information about the studied material such as chemical composition, crystallographic structure, and physical properties. Also, images of the NPs interacted with the packaging materials can be obtained by SEM and TEM. Besides, FTIR is used to reveal the chemical changes after NP incorporation.

#### Intelligent Packaging and Smart Packaging

Intelligent packaging has intelligent functions, such as sensing, detecting, tracing, recording, and communicating [140]. This system utilizes a number of indicators for monitoring the food quality in terms of microbial growth as well as temperature and packing integrity [144, 145], whereas smart packaging possesses the susceptibilities of intelligent and active packaging.

## Conclusions and Future Perspective

This current review aimed to discuss and analyze research works that addressed the potential use of ZnO-NPs for antibacterial activity. Extensive discussion was centered on the antibacterial activity of ZnO-NPs coupled with a number of influenced factors impacting the activity. Mainly, by improving factors like UV illumination, ZnO particle size, concentration, morphology, and surface modification, powerful antibacterial results would be obtained. These factors influence a variety of toxicity mechanisms. Special focus was given to mechanisms of action which come as the hottest issue in the antibacterial activity. The induction of intracellular ROS generation can cause death and have been considered as a major ability of ZnO-NPs. Release of Zn^2+^ ions and adhesion on the cell membrane cause mechanical damage to the cell wall. Additionally, a brief presentation of a study conducted by authors of this review was explored. Finally, a concise discussion was given to one vital application as antimicrobial agent on food.

The importance and significance of ZnO-NPs in various areas has developed global interest to study their antibacterial activity. The documented antibacterial actions of ZnO-NPs have stimulated a considerable range of antimicrobial applications. ZnO-NPs possess unique properties and excellent stability with long life compared with organic-based disinfectants that stimulated its use as antibacterial agent. The large surface area-to-volume ratio allows their use as novel antimicrobial agents, which are coming up as recent concern for researchers.

A goal of this review is to set a well-built reference for scientists interested in antibacterial activities along with their functional applications by considering nanotechnology principles as it relates to the nanobiological toxicity of ZnO-NPs. The noble properties and attractive characteristics of ZnO-NPs confer significant toxicity to organisms, which have made ZnO-NPs successful candidate among other metal oxides. Other specific properties are predicted to expand ZnO-NPs applications in several areas, particularly in catalysis and biomedicine. A number of significant breakthroughs have emerged in the areas of antimicrobial applications, as in the food industry.

This survey revealed the sensitivity of ZnO-NPs toward characteristic microorganisms that are of threatening concern. Based on the toxicity mechanism of ZnO-NPs, this review concludes that the toxicity differs from one study to another according to the test conditions, further mechanisms and researches are currently being investigated. Additional research is required to investigate the exact toxicity mechanisms to deeply elucidate the sensitivity of bacteria to ZnO-NPs, as the results to date are quite promising. However, this will necessitates further researches to adequately scrutinize the NPs properties. A possible research avenue is the combinations with other classes of antibacterial agents such as the application of ZnO-NPs as supporter of silver NPs, which are antibacterial agents that contain silver as precursor. This topic is regarded as a powerful application forecast and marketable significance.

More emphasis should be given to the correlation between ZnO-NPs structural, optical, electrical, chemical properties, and their bacterial toxicity. ZnO-NPs can act as smart weapon toward multidrug-resistant microorganisms and a talented substitute approach to antibiotics. The toxicological influence of ZnO-NPs should be evaluated to determine the consequences of using these NPs in food safety. It is anticipated that this review may be able to enhance further research into novel methodological characterization and clinical correlations in this topic. Meanwhile, solutions would be suggested to consequences of health-related problems by addressing this complex through research and scientific reports.

## Abbreviations

ROS: Reactive oxygen species
MIC: The minimum inhibitory concentration
MBC: Minimum bactericidal concentration
XRD: X-ray diffraction
FESEM: Field emission scanning electron microscope
TEM: Transmission electron microscope
EDX: Energy dispersive X-ray spectroscopy
ESI: Electron spectroscopy imaging, by energy-filtered transmission electron microscopy (EFTEM)
IV: Current–voltage measurement
ATCC: American Association of Textile Chemists and Colorists
NA: Nutrient agar
LB: Luria–Bertani broth
TSB: Trypticase soy broth
TSA: Tryptic soy agar
BA: Blood agar
CFU: Colony forming unit
PEG: Polyethylene glycol
PVP: Polyvinylpyrrolidone
PGA: Poly(α, γ, l-glutamic acid)
